# Supplementary material for: Measuring the counterion cloud of soft microgels using SANS with contrast variation
Source: Nat Commun. 2023 Jul 7;14:3827. doi: 10.1038/s41467-023-39378-5 (PMC10329000; doi:10.1038/s41467-023-39378-5)
Supplement: Supplementary file 1 — Supplementary Information [file 41467_2023_39378_MOESM1_ESM.pdf]

Supplementary Information: Measuring the counterion cloud of soft microgels using SANS with contrast variation

Boyang Zhou[1], Urs Gasser[1*†], and Alberto Fernandez-Nieves[2,3,4]

[1]Laboratory for Neutron Scattering and Imaging, Paul Scherrer Institut, Forschungsstrasse 111, 5232, Villigen, Switzerland.
[2]Department of Condensed Matter Physics, University of Barcelona, Carrer de Martí i Franquès 1, Barcelona, 08028, Spain.
[3]ICREA-Institucio Catalana de Recerca i Estudis Avançats,Barcelona, 08028, Spain.
[4]Institute for Complex Systems (UBICS), University of Barcelona, Barcelona, 08028, Spain.
[*]Address correspondence to: urs.gasser@psi.ch
[1]Contrinuting authors: boyang.zhou@psi.ch; a.fernandeznieves@ub.edu;
[†]B.Z. and U.G. contributed equally to this work.

1 Microgel suspensions

We study three temperature-sensitive pNIPAM microgels with different swollen radii containing 2.7 wt% of crosslinker N'-methylene-bis-acrylamide (BIS). Ammonium persulfate [APS, $(NH_4)_2S_2O_8$] is used as the initiator of the polymerization reaction. Charged –O–SO_2–O^- groups remain in the pNIPAM network and are assumed to be located in the periphery of the microgel, while NH_4^+ are present as counterions. The SO_4^- groups originating from the APS attack a carbon-carbon double bond to form a radical O_3S–O–C–C(.), where the (.) symbol indicates the radical is centered on the distal carbon. The end-group structure formed by the sulfate, therefore, looks like a sulfonic acid with an O–C bond attaching it to the growing polymer. Therefore, the charged group is –SO_2–O^- and not SO_4^- [1]. The surfactant sodium dodecyl sulfate [SDS, $CH_3(CH_2)_{11}OSO_3Na$] is used to control the microgel size and to obtain monodisperse particles; Na^+ ions are therefore also present as counterions. After the synthesis, the microgel suspension is cleaned to remove any unreacted monomers and the SDS. The cleaned, final suspension of pNIPAM microgels thus contains Na^+ and NH_4^+ counterions. Most of these are electrostatically bound to the microgels.

We count counterions with electrostatic potential energy $|e\psi| > 1.5 k_{\mathrm{B}}T$ as bound to the microgel. These ions are mobile within the ion cloud but cannot escape to explore the whole suspension volume. Our Poisson-Boltzmann calculations using the cell model [2] allow us to estimate the fraction of

bound counterions to be about 98% at $\phi = 0.1$ and 89% at $\phi = 0.5$. The bound counterions cannot leave the counterion cloud at the periphery of the microgel, while the remaining counterions are essentially free, bound with a strength $\lesssim k_{\mathrm{B}}T$, and can thus explore all of the suspension volume; these ions, therefore, contribute to the osmotic pressure.

Besides temperature, the suspension behavior is controlled by the volume fraction ϕ, which can vary in a microgel suspension in non-trivial ways, as microgels may deswell and interpenetrate when sufficiently concentrated. Therefore, we use a generalized volume fraction

$$\zeta = \frac{N_{\mathrm{tot}} V}{V_{\mathrm{tot}}} \approx \frac{m_{\mathrm{pNIPAM}}}{m_{\mathrm{tot}}} \frac{\rho_{\mathrm{solvent}}}{\rho_{\mathrm{pNIPAM}}} \frac{R_{\mathrm{sw}}^3}{R_{\mathrm{coll}}^3} \tag{1}$$

where V is the microgel volume in the fully swollen state, and N_{tot}, V_{tot}, m_{pNIPAM}, and m_{tot} are the number of particles, the suspension volume, the mass of pNIPAM polymer, and the mass of the suspension, respectively. The density of the polymer is $\rho_{\mathrm{pNIPAM}} = 1.269$ g/cm^3 [3] and that of the suspension is assumed to be the same as the density of the H_2O or D_2O solvent given the polymer concentration is low. The radii R_{sw} and R_{coll} correspond to the swollen and collapsed states, respectively. The latter is the radius of the dry microgel without any solvent.

The swollen radius is obtained using dynamic light scattering (DLS). For DLS, samples are prepared in Milli-Q H_2O with $\zeta < 0.01$. This is dilute enough for microgel-microgel interactions to be negligible. Hence, the single-particle diffusion coefficient, D, can readily be obtained from the time-correlation function of the scattered intensity. We then use the Stokes-Einstein equation, $D = \frac{k_{\mathrm{B}}T}{6\pi\eta_{\mathrm{s}}R_{\mathrm{h}}}$, with k_{B} the Boltzmann constant, T the absolute temperature and η_{s} the solvent viscosity, to obtain the hydrodynamic radius, R_h, which corresponds to R_{sw} [4].

The collapsed radius R_{coll} in Eq. 1 is obtained via viscosimetry. We measure the suspension viscosity, η, as a function of the polymer mass fraction in the suspension, $c = m_{\mathrm{pNIPAM}}/m_{\mathrm{tot}}$, and use the Einstein-Batchelor relation [5] for the relative viscosity,

$$\begin{aligned} \eta_r &= 1 + 2.5\zeta + 5.9\zeta^2 \\ &= 1 + 2.5(kc) + 5.9(kc)^2, \end{aligned} \tag{2}$$

to obtain the proportionality constant

$$k = \frac{\rho_{\mathrm{solvent}} R_{\mathrm{sw}}^3}{\rho_{\mathrm{pNIPAM}} R_{\mathrm{coll}}^3} \tag{3}$$

between ζ and the mass ratio $c = m_{\mathrm{pNIPAM}}/m_{\mathrm{tot}}$. From k, we obtain R_{coll}, using R_{sw} obtained with DLS:

$$R_{\mathrm{coll}} = \left(\frac{\rho_{\mathrm{solvent}}}{\rho_{\mathrm{pNIPAM}}} \frac{R_{\mathrm{sw}}^3}{k} \right)^{1/3}. \tag{4}$$

The radii R_{sw} and R_{coll}, as well as the conversion constant k, are reported in Supplementary Tab. 1.

To learn about the counterion cloud of the pNIPAM microgels, we prepare two samples for each microgel, one with sodium, Na^+, and the other one with ammonium, NH_4^+, counterions. To exchange the counterions, we prepare dilute microgel suspensions in Milli-Q H_2O, introduce them

in dialysis bags, and place them in solutions of NaCl or NH_4Cl at a concentration of (167 ± 5) mM to allow for counterion exchange. The salt solutions are exchanged daily for 5 days to obtain a full exchange of ions. The samples are then dialyzed against ultrapure Milli-Q H_2O for another 5 days to remove the salt from the microgel suspensions. After the dialysis, we freeze dry the suspensions to obtain the dry microgel powder, which we finally re-suspend in D_2O. After freeze drying, about one H_2O molecule per NIPAM monomer is expected to remain in the pNIPAM powder [6]. The resultant contamination after resuspension in D_2O is thus expected to be < 1 wt%. This implies a $< 1\%$ reduction in the scattering length density of the solvent, which does not affect our analysis and conclusions.

Sample	$R_{\rm sw}$ (nm)	$R_{\rm coll}$ (nm)	k (at 20°C)
s1	87 ± 1.7	25 ± 0.6	24.78 ± 0.004
s2	125 ± 2.7	38 ± 0.8	20.56 ± 0.005
s3	140 ± 2.3	40 ± 0.8	18.74 ± 0.005

Supplementary Table 1: The swollen hydrodynamic radius $R_{\rm sw}$ measured with DLS at 20 °C and the collapsed radius $R_{\rm coll}$ calculated with Eq. 4 from the conversion constant k obtained with viscosimetry at 20 °C for the three used microgel samples.

2 SANS data analysis

SANS data of all our samples was taken at $T \approx 20\,^{\circ}\mathrm{C} < T_{\rm LCST}$ and $T \approx 45\,^{\circ}\mathrm{C} > T_{\rm LCST}$ to investigate microgels with their Na^+ or NH_4^+ ion clouds in both the fully swollen and the deswollen states. Data was taken to obtain $\sim 2 \cdot 10^6$ counts on the detector to have good statistics in the q range of interest. All scattering curves are fitted with a model including the fuzzy-sphere form factor [7], the Percus-Yevick structure factor, $S(q)$, with polydispersity, and the q resolution of the SANS instrument. The Percus-Yevick structure factor has been found to be adequate for microgel suspensions [8]. The polydispersity of pNIPAM suspensions is typically close to 10% [9]. Hence we use the so-called apparent structure factor [10]

$$S'(q) = 1 + \beta(q)[S(q) - 1], \tag{5}$$

where $\beta(q) = |\langle F_{\rm p}(q)\rangle|^2 / \langle |F_{\rm p}(q)|^2 \rangle$ is a factor varying between zero and one that suppresses the oscillations of the monodisperse structure factor $S(q)$ and, therefore, approximates the effect of polydispersity. The assumption behind this approach is that the microgel size is not correlated with the structure of the suspension. This is a good approximation for modest polydispersities $\sim 10\%$ and for moderate concentrations, $\zeta \lesssim 0.5$. If the polydispersity is significant and the suspension is concentrated, one should obtain the pair structure factors by solving the matrix form of the Ornstein-Zernike equation for the pair potential [11]. Using the apparent structure factor $S'(q)$, the scattering intensity becomes

$$I(q) = n_{\rm d} \langle V_{\rm c}^2 \Delta\rho_{\rm c}^2 \rangle P(q) S'(q). \tag{6}$$

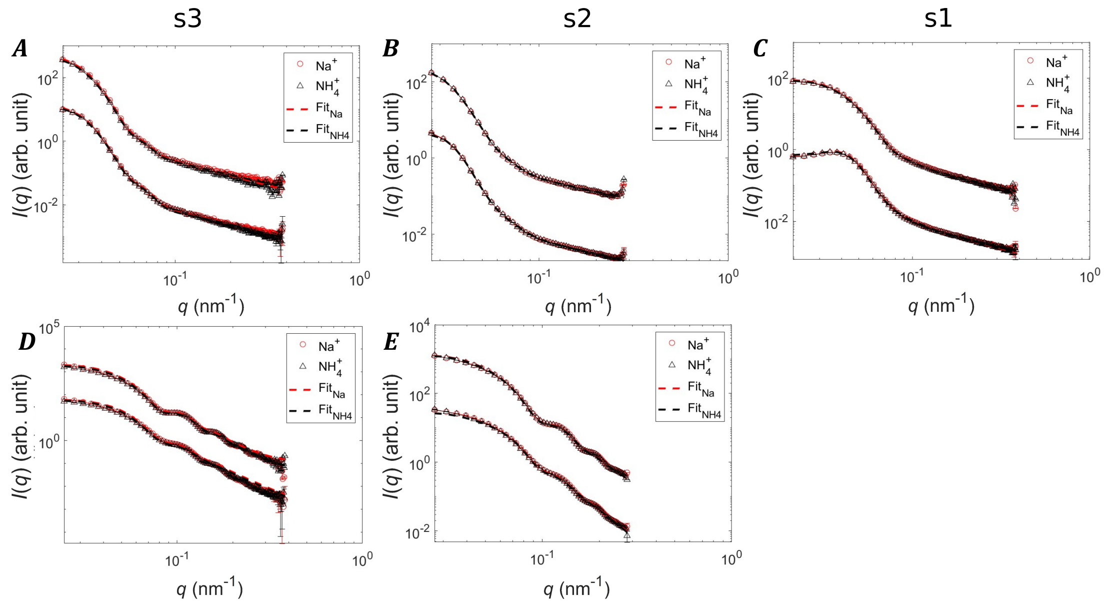

Supplementary Figure 1: Simultaneous fits (−−) to SANS measurements with Na^+ (○) and NH_4^+ (△) counterions for all samples: top curves are for $0.1 \lesssim \zeta \lesssim 0.2$ and bottom curves are for $0.48 \lesssim \zeta \lesssim 0.55$. A: sample s3 at 20 °C. B: sample s2 at 20 °C. C: sample s1 at 20 °C. D: sample s3 at 45 °C. E: sample s2 at 45 °C. Data from samples with $0.1 \lesssim \zeta \lesssim 0.2$ (upper curves) has been multiplied by 10^3 for the sake of clarity. The error bars represent the uncertainty due to the counting statistics of the SANS instrument.

For the form factor $P(q) = \langle |F_\mathrm{p}(q)|^2 \rangle / \langle |F_\mathrm{p}(0)|^2 \rangle$, we use the fuzzy sphere model [12]

$$F_\mathrm{p}(q) \quad = \quad V_\mathrm{c} \Delta\rho_\mathrm{c} \frac{3\left[\sin(qR_\mathrm{c}) - qR_\mathrm{c}\cos(qR_\mathrm{c})\right]}{(qR_\mathrm{c})^3} \exp\left(-\frac{q^2\sigma_\mathrm{s}^2}{2}\right), \tag{7}$$

which is well-accepted for microgels. In this expression, $V_\mathrm{c} = 4\pi R_\mathrm{c}^3/3$ is the volume of the core, and $\Delta\rho_\mathrm{c}$ is the scattering-length-density contrast between the microgel core and the pure solvent. The prefactor $V_\mathrm{c}\Delta\rho_\mathrm{c}$ can also be expressed using the contrast of pure pNIPAM and the solvent, $\Delta\rho_\mathrm{p} = \rho_\mathrm{p} - \rho_\mathrm{s}$, if the pNIPAM volume, V_p, is used as the volume of the scatterer: $V_\mathrm{c}\Delta\rho_\mathrm{c} = V_\mathrm{p}\Delta\rho_\mathrm{p}$. In real space, Eq. 7 corresponds to a spherical core of radius R_c and a constant scattering-length density that is convolved with a Gaussian with standard deviation σ_s to obtain the fuzzy corona. The width of the corona is $2\sigma_\mathrm{s}$ and the radius of the particle seen by SANS is $R_\mathrm{SANS} = R_\mathrm{c} + 2\sigma_\mathrm{s}$. The size polydispersity is modeled with a log-normal distribution for the core radius, which is used for microgels and polydispersities that are too high to be modeled with a Gaussian distribution [13]:

$$D(x) = \frac{1}{x}\frac{1}{\sqrt{2\pi}\sigma} \exp\left[-\frac{(\log x - \mu)^2}{2\sigma^2}\right], \tag{8}$$

where σ is the parameter setting the polydispersity $\left\langle (x - \langle x \rangle)^2 \right\rangle = \langle x \rangle^2 (e^{\sigma^2} - 1)$ and the mean is $\langle x \rangle = e^{\mu + \frac{\sigma^2}{2}}$. Inhomogeneities within the microgel contribute to the form factor and are taken into account with a Lorentzian term $I_\mathrm{chain}(q) = I_\mathrm{chain}(0)/[1 + (\xi q)^2]$, where ξ is the correlation length of

the polymer network and $I_{\rm chain}(0)$ is the zero-q intensity due to these inhomogeneities [14, 15]. This signal dominates the form factor for $q \gtrsim \pi/\sigma_{\rm s}$. With this, the average form factor is

$$P(q) \;\; = \;\; \frac{1}{\langle V_{\rm c}^2 \Delta\rho_{\rm c}^2 \rangle} \int_0^\infty D(R_{\rm c}) |F_{\rm p}(q;R_{\rm c})|^2 dR_{\rm c} + I_{\rm chain}(q) \tag{9}$$

with the normalization factor $\langle |F_{\rm p}(0)|^2 \rangle = \langle V_{\rm c}^2 \Delta\rho_{\rm c}^2 \rangle = \int_0^\infty V_{\rm c}^2(R_{\rm c})\, \Delta\rho_{\rm c}^2\, D(R_{\rm c})\, dR_{\rm c}$. Lastly, the smearing effect due to instrument resolution is considered by convolving $I(q)$ with a Gaussian [16]:

$$I_{\rm s}(q) = \frac{1}{\sqrt{2\pi}\sigma_{\rm r}(q)} \int_0^\infty \exp\left[-\frac{(q-q')^2}{2\sigma_{\rm r}^2(q)} \right] I(q')\, dq' + B, \tag{10}$$

where the constant B accounts for the background due to incoherent scattering. We use this model to fit the scattering signal of our microgel suspensions to extract the particle size, the particle number density, and the polydispersity, which are listed in Supplementary Tab. 2. We find that the microgel size and internal structure do not depend on the counterion species. The number density appears as a parameter for the structure factor $S'(q)$; it is through this dependence that we can extract $n_{\rm d}$ from the SANS curve. Recall $n_{\rm d}$ also appears as a prefactor in $I(q)$, because the scattered intensity depends on the number of microgels present in the suspension, see Eq. 6.

3 Counterion cloud model

As the scattering signal originating from the ion cloud is small compared to the total measured signal, we amplify the signal strength by subtracting $I^{\rm Na^+}(q)$ from $I^{\rm NH_4^+}(q)$ after correcting for small differences in $n_{\rm d} \cdot S'(q)$:

$$\begin{aligned} \Delta I(q) \;\; &= \;\; I^{\rm NH_4^+}(q) - I^{\rm Na^+}_{\rm corr}(q) \\ &= \;\; n_{\rm d}\, \Delta\rho_{\rm c}^2\, V_{\rm c}^2 \left(P^{\rm NH_4^+}(q) - P^{\rm Na^+}(q) \right) S'(q), \end{aligned} \tag{11}$$

where the prefactor includes the number density $n_{\rm d}$, the particle scattering contrast $\Delta\rho_{\rm c}$, and the core volume $V_{\rm c}$. Furthermore, we consider the contribution of the counterion cloud to the form factor, $F_{\rm ic}(q)$, in addition to the polymer contribution, $F_{\rm p}(q)$, that is modeled with Eq. 7:

$$\left[F_{\rm p}(q) + F_{\rm ic}^X(q) \right]^2 \;\; = \;\; F_{\rm p}^2(q) + 2F_{\rm p}(q) F_{\rm ic}^X(q) + F_{\rm ic}^{X\,2}(q). \tag{12}$$

With this, we rewrite the difference between the signals of the samples with NH_4^+ and Na^+ counterions:

$$\Delta I_{\rm m}(q) \approx 2\, n_{\rm d} F_{\rm p}(q) \left[F_{\rm ic}^{\rm NH_4^+}(q) - F_{\rm ic}^{\rm Na^+}(q) \right] S'(q), \tag{13}$$

where we have assumed that the terms $\propto F_{\rm ic}^2(q)$ are negligible due to the low counterion density. The subscript ‘m’ indicates that the scattering intensity is given for the monodisperse case with fixed microgel size; polydispersity will be included below in Eq. 18. $\Delta I_{\rm m}(q)$ is directly proportional to the

difference between the scattering amplitudes $F_{\rm ic}^X(q)$ of the clouds, which we split into an ion-specific prefactor and an ion-independent structural part:

$$F_{\rm ic}^X(q) = \Delta\rho^X V_{\rm ic}^X\, f_{\rm ic}(q), \tag{14}$$

where $\Delta\rho^X$ is the scattering-length density contrast of the ion with respect to the solvent and $V_{\rm ic}$ is the volume taken by the ions, which is given by the product of the number of ions and the volume of a single ion. We estimate that there are approximately $7 \cdot 10^4$ counterions per microgel [17, 18]. For the ion volumes, we use the ion radii for Na^+, 0.116 nm, and for NH_4^+, 0.146 nm, as reported in the literature.

As the $–O–SO_2–O^-$ groups at the ends of the pNIPAM chains are expected to be located in the periphery of the microgel, we model the counterion cloud as a smeared-out spherical surface. We use a convolution with a Gaussian with standard deviation $\sigma_{\rm ic}$ to obtain the smearing:

$$f_{\rm ic}(q) = \frac{\sin(qR_{\rm ic})}{qR_{\rm ic}}\, \exp\left(-\frac{q^2\sigma_{\rm ic}^2}{2}\right), \tag{15}$$

where the cloud radius is given by the core radius of the microgel, $R_{\rm c}$, and an offset Δr, such that $R_{\rm ic} = R_{\rm c} + \Delta r$. This links the cloud radius to the structure of the pNIPAM network of the microgel and implies that the polydispersity of the pNIPAM core is also applied to the counterion cloud. By inserting Eq. 14 into Eq. 13, we have

$$\Delta I_{\rm m}(q) \quad \approx \quad 2\, n_{\rm d}\left(\Delta\rho^{\rm NH_4^+} V_{\rm ic}^{\rm NH_4^+} - \Delta\rho^{\rm Na^+} V_{\rm ic}^{\rm Na^+}\right) F_{\rm p}(q)\, f_{\rm ic}(q)\, S'(q). \tag{16}$$

In this difference, the counterion cloud signal, $f_{\rm ic}(q)$, is amplified by the stronger signal from the polymer, $F_{\rm p}(q)$. The magnitude is determined by the scattering-length-density contrast between NH_4^+ and Na^+. As $\rho^{\rm NH_4^+} \approx -4.3 \cdot 10^{-6}$Å^{-2} and $\rho^{\rm Na^+} \approx 5.6 \cdot 10^{-6}$Å^{-2}, the contrast is significant and strengthens the signal originating from the cloud.

Our analysis of the scattering curves indicates that samples are indeed prepared at very similar concentrations and can be expected to have the same structure factor. The exchange of counterions using dialysis does not appear to affect the contribution of the pNIPAM polymer to the form factor, as shown by the structural parameters listed in Supplementary Tab. 2.

Combining Eqs. 7 and 15 with Eq. 16, we obtain the model for the scattering-intensity difference:

$$\begin{aligned}\Delta I_{\rm m}(q) \quad \approx \quad & 2\, n_{\rm d}\, \Delta\rho_{\rm c} V_{\rm c}\left(\Delta\rho^{\rm NH_4^+} V_{\rm ic}^{\rm NH_4^+} - \Delta\rho^{\rm Na^+} V_{\rm ic}^{\rm Na^+}\right) \\ & \times \frac{3\left[\sin(qR_{\rm c}) - qR_{\rm c}\cos(qR_{\rm c})\right]}{(qR_{\rm c})^3}\, \frac{\sin(qR_{\rm ic})}{qR_{\rm ic}} \\ & \times \exp\left[-\frac{q^2\left(\sigma_{\rm ic}^2 + \sigma_{\rm s}^2\right)}{2}\right] S'(q), \end{aligned} \tag{17}$$

which is then convolved with the log-normal distribution to account for the polydispersity of $R_{\rm c}$, as

			Individual fit					Simultaneous fit			
Sample	ζ ± 0.04	T (°C) ± 0.5	R_c (nm)	σ_s (nm)	R_{SANS} (nm)	ξ (nm)	σ(%)	R_{SANS} (nm)	n_d^X (nm^{-3}) $\times 10^{-8}$	n	Figs.
s1 Na^+	0.10	20	55.6 ± 0.5	14.9 ± 0.3	85.4 ± 1.1	12 ± 1	15 ± 1.0	83.9 ± 1.5	3.43 ± 0.18	0.95 ± 0.07	S1(C)
s1 NH_4^+	0.09	20	56.0 ± 0.4	14.3 ± 0.5	84.6 ± 1.4	12 ± 1	15 ± 1.0	"	3.27 ± 0.18	"	S2(C)
s1 Na^+	0.47	20	50.9 ± 0.5	13.5 ± 0.4	77.9 ± 1.3	12.4 ± 1	15 ± 1.0	78.8 ± 0.8	17.22 ± 0.58	0.96 ± 0.04	S1(C)
s1 NH_4^+	0.46	20	51.0 ± 0.5	14.2 ± 0.3	79.4 ± 1.1	12.9 ± 1	15 ± 0.9	"	16.49 ± 0.58	"	S2(H)
s2 Na^+	0.11	20	82.0 ± 0.7	21.1 ± 0.2	124.2 ± 1.1	14.5 ± 1	10 ± 0.5	124.4 ± 1.7	1.33 ± 0.06	1.01 ± 0.06	S1(B)
s2 NH_4^+	0.11	20	82.1 ± 0.5	21.2 ± 0.4	124.5 ± 1.3	14 ± 1	11 ± 0.5	"	1.34 ± 0.06	"	S2(B)
s2 Na^+	0.48	20	80.6 ± 0.5	19.3 ± 0.4	119.2 ± 1.3	9.2 ± 1	13 ± 0.7	118.7 ± 1.2	5.91 ± 0.19	0.99 ± 0.05	S1(B)
s2 NH_4^+	0.47	20	81.3 ± 1.1	18.7 ± 0.4	118.7 ± 1.9	8.6 ± 1	12 ± 0.5	"	5.82 ± 0.19	"	S2(G)
s2 Na^+	0.11	45	46.7 ± 0.3	2.4 ± 0.7	51.4 ± 1.3	N/A	11 ± 0.9	49.5 ± 1.3	1.38 ± 0.05	0.95 ± 0.08	S1(E)
s2 NH_4^+	0.11	45	46.7 ± 0.3	2.2 ± 0.7	51.2 ± 1.3	N/A	11 ± 1.0	"	1.30 ± 0.07	"	S2(E)
s2 Na^+	0.48	45	46.7 ± 0.5	2.8 ± 0.7	52.3 ± 1.9	N/A	13 ± 0.5	49.4 ± 1.2	5.94 ± 0.29	0.94 ± 0.06	S1(E)
s2 NH_4^+	0.46	45	46.7 ± 0.3	2.7 ± 0.8	52.2 ± 1.9	N/A	13 ± 0.5	"	5.54 ± 0.21	"	S2(J)
s3 Na^+	0.18	20	87.5 ± 1.0	24.1 ± 0.8	135.6 ± 2.6	14 ± 1	11 ± 0.6	134.3 ± 1.6	1.57 ± 0.06	0.93 ± 0.05	1, 2, S1(A),
s3 NH_4^+	0.17	20	87.9 ± 1.0	23.9 ± 0.8	135.7 ± 2.6	14 ± 1	11 ± 0.6	"	1.45 ± 0.06	"	S2(A), S3, S4
s3 Na^+	0.56	20	88.2 ± 0.8	21.8 ± 0.3	131.8 ± 1.4	8.6 ± 1	10 ± 0.4	128.8 ± 1.8	4.89 ± 0.21	0.94 ± 0.06	S1(A)
s3 NH_4^+	0.53	20	88.6 ± 0.8	21.3 ± 0.4	131.2 ± 1.6	9.0 ± 1	9 ± 0.6	"	4.61 ± 0.20	"	S2(F)
s3 Na^+	0.18	45	54.4 ± 1.3	0 ± 0.01	54.4 ± 1.3	N/A	12 ± 0.9	54.61 ± 0.8	1.62 ± 0.08	0.92 ± 0.06	1
s3 NH_4^+	0.17	45	54.5 ± 1.2	0 ± 0.01	54.5 ± 1.2	N/A	12 ± 1.0	"	1.49 ± 0.06	"	S1(D)
s3 Na^+	0.57	45	55.4 ± 0.9	0 ± 0.01	55.4 ± 0.9	N/A	12 ± 0.8	55.24 ± 0.7	4.96 ± 0.19	0.91 ± 0.05	1, S2(D)
s3 NH_4^+	0.52	45	55.6 ± 1.1	0 ± 0.01	55.6 ± 1.1	N/A	12 ± 0.8	"	4.53 ± 0.18	"	S1(D), S2(I)

Supplementary Table 2: Results from individual SANS fits (Eq. 10) for all samples and from simultaneous fits for sample pairs. The sample name with ion species, the generalized volume fraction (ζ) obtained from the fit, and the temperature of the SANS measurement are given to identify sample and measurement. For individual fits, the core radius (R_c), corona width (σ_s), SANS radius (R_{SANS}), and polydispersity (σ) are listed. For simultaneous fits, the SANS radius (R_{SANS}), the microgel number density (n_d^X), and the number-density ratio ($n = n_d^{NH_4^+}/n_d^{Na^+}$) are given. In the last column, the figures displaying the respective measurement and fit are indicated.

also done in Eq. 9:

$$\Delta I(q) = \int_0^\infty D(R_\mathrm{c})\, \Delta I_\mathrm{m}(q)\, dR_\mathrm{c}. \tag{18}$$

Note that the microgel polydispersity is also used for the counterion cloud, as R_ic is defined to depend on the core radius R_c. We also emphasize that since $S'(q)$ already accounts for polydispersity, it is taken out of the R_c average above to avoid over-considering polydispersity effects in the suspension structure factor. Finally, we consider the instrument resolution by integrating $\Delta I(q)$ with a Gaussian distribution, analogous to Eq. 10:

$$\Delta I_\mathrm{s}(q) \quad = \quad \frac{1}{\sqrt{2\pi}\sigma_\mathrm{r}(q)} \int_0^\infty \exp\left(-\frac{[q-q']^2}{2\sigma_\mathrm{r}^2(q)}\right) \Delta I(q')dq'. \tag{19}$$

Using this model, we extract the key parameters R_ic and σ_ic of the counterion cloud. The number density, n_d, the core radius, R_c, the width of the corona, σ_s, and the polydispersity, σ, also enter. The dependence of the model given by Eq. 19 on the parameters is illustrated in Fig. 2C, D of the main text.

4 Counterion cloud fits

The general steps of data preparation before fitting with the cloud model (Eqs. 17, 18 and 19) are mentioned in the main text. To correct for differences in concentration, we assume that the counterion species, Na^+ or NH_4^+, do not change the microgel architecture given by R_c and σ_s, the polydispersity (σ), and the correlation length (ξ); only the number density is expected to slightly differ for samples containing Na^+ and NH_4^+ counterions. We perform a simultaneous fit with the model given by Eq. 10 using as common free parameters R_c, σ_s, σ and ξ, and then the two number densities, $n_\mathrm{d}^{\mathrm{Na}^+}$ and $n_\mathrm{d}^{\mathrm{NH}_4^+}$. The assumption is that the small concentration difference does not cause a change in the form factor or the structure factor; this is supported by our results obtained with individual fits, see Supplementary Tab. 2. The simultaneous fits shown in Supplementary Fig. 1 confirm that these fits and the extracted number densities are in good agreement with the raw data. The number density n_d^X (X = Na^+ or NH_4^+) for each set of data is summarized in Supplementary Tab. 2. We then calculate the number density ratio, $n = n_\mathrm{d}^{\mathrm{NH}_4^+}/n_\mathrm{d}^{\mathrm{Na}^+}$ and correct $I^{\mathrm{Na}^+}(q)$ as $I_\mathrm{corr}^{\mathrm{Na}^+}(q) = n\, I^{\mathrm{Na}^+}(q)$. For each Na^+, NH_4^+ pair, the correction factor n is expected to be the same for the measurements taken below and above T_LCST, as the number density n_d does not depend on temperature. This is the case within the error limits, see Supplementary Tab. 2.

In Supplementary Fig. 3, we show the corrected $I_\mathrm{corr}^{\mathrm{Na}^+}(q)$ together with $I^{\mathrm{NH}_4^+}(q)$. As the correction is small, we can barely notice any difference with respect to the uncorrected data shown in Fig. 1 (main text). But the number-density correction is important to reveal the signal due to the counterions.

After the correction, we fit all the number-density-corrected $\Delta I(q)$ data with the model introduced in Eqs. 17, 18, and 19. The results for all samples are summarized in Supplementary Tab. 3, Fig. 3 (main text), and Supplementary Fig. 5. The radius of the cloud, R_ic, is always smaller but

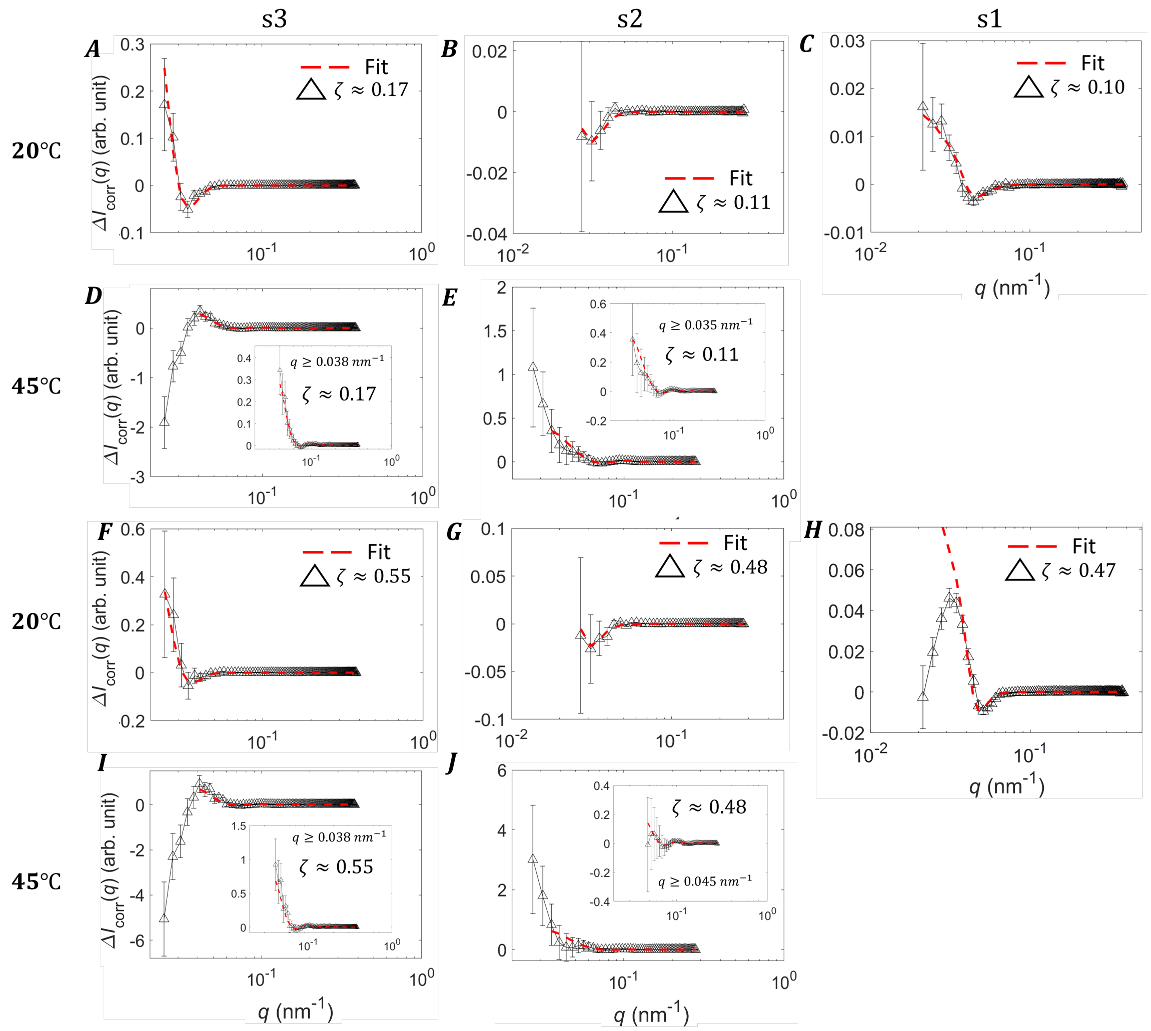

Supplementary Figure 2: $\Delta I(q)$ and counterion cloud fits for the samples s3 (1st column, panels A, D, F, I), s2 (2nd column, panels B, E, G, J), and s1 (3rd column, panels C, H). The temperature of the SANS measurements is given on the left for each row. Samples with $0.1 \lesssim \zeta \lesssim 0.2$ are shown in the two upper rows (panels A-E), and the two lower rows (panels F-J) are for $0.48 \lesssim \zeta \lesssim 0.55$. The inserts in panels D, E, I, and J are magnifications to show the counterion cloud signal more clearly. The error bars represent the uncertainty due to the counting statistics of the SANS instrument.

close to R_{SANS}, which implies that the fixed charges are located in the outskirts of the microgel. The width of the cloud, σ_{ic}, is found to be comparable to the width of the fuzzy shell, $2\sigma_{\mathrm{s}}$, and is in good agreement with our previous estimate, (35 ± 4) nm [9, 17]. When we average σ_{ic} for all samples measured at $T \approx 20\,^{\circ}\mathrm{C}$ listed in Supplementary Tab. 3, we get $\langle \sigma_{\mathrm{ic}} \rangle = (38 \pm 8)$ nm.

We compare the intensity of the observed signal due to the ions with the expected intensity given by the prefactors of the form factor of the polymer network and the counterion cloud, which are a product of the volume and the contrast of the scatterer, see Eq. 17. For the polymer network, we use that $\Delta\rho_{\mathrm{c}} V_{\mathrm{c}} = \Delta\rho_{\mathrm{p}} V_{\mathrm{p}}$ and take the scattering-length densities of pure NIPAM, $\rho_{\mathrm{p}} = (0.939 \pm$

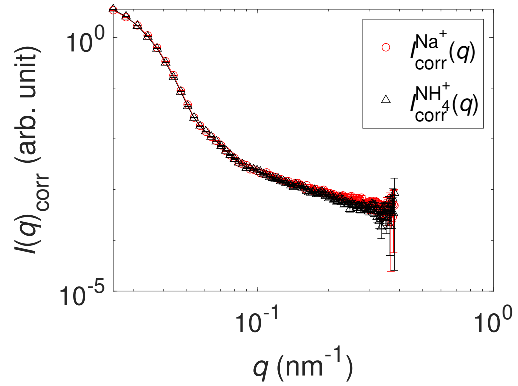

Supplementary Figure 3: Number-density corrected $I_{\mathrm{corr}}^{\mathrm{Na}^+}(q) = n\, I^{\mathrm{Na}^+}(q)$ with n = 0.925 and the uncorrected $I^{\mathrm{NH}_4^+}(q)$ for sample s3 at $\zeta \approx 0.2$. The error bars represent the uncertainty due to the counting statistics of the SANS instrument.

$0.001) \cdot 10^{-4}\,\mathrm{nm}^{-2}$, and the D_2O solvent, $\rho_{\mathrm{D_2O}} = (6.335 \pm 0.001) \cdot 10^{-4}\,\mathrm{nm}^{-2}$ to obtain the contrast $\Delta\rho_{\mathrm{p}} = \rho_{\mathrm{p}} - \rho_{\mathrm{D_2O}} = (-5.497 \pm 0.002) \cdot 10^{-4}\,\mathrm{nm}^{-2}$. With this contrast, the volume of the scatterer is that of the pure polymer, which is given by the collapsed radius of the microgel, $V_{\mathrm{p}} = (4\pi/3)R_{\mathrm{coll}}^3$ with R_{coll} given in Supplementary Tab. 1. For the counterion cloud, we use the tabulated scattering lengths of H, N, and Na and the ion radii given above to obtain the contrasts $\Delta\rho^{\mathrm{NH_4}} = \rho^{\mathrm{NH_4}} - \rho_{\mathrm{D_2O}} = (-10.6 \pm 0.1) \cdot 10^{-4}\,\mathrm{nm}^{-2}$ and $\Delta\rho^{\mathrm{Na}} = \rho^{\mathrm{Na}} - \rho_{\mathrm{D_2O}} = (-0.78 \pm 0.01) \cdot 10^{-4}\,\mathrm{nm}^{-2}$. The number of counterions per microgel, $N_{\mathrm{i}} = (7 \pm 1) \cdot 10^4$, estimated from particle synthesis [17], is used to obtain the total volume taken by the ions in the cloud, $V_{\mathrm{ic}} = (4\pi/3)R_X^3 N_{\mathrm{i}}$ with the ion radius R_X and X representing either Na^+ or NH_4^+. The prefactor of the form factor of a microgel including the counterion cloud, therefore, is $s = \left(V_{\mathrm{p}}\Delta\rho_{\mathrm{p}} + V_{\mathrm{ic}}\Delta\rho^X\right)^2 \approx \left(V_{\mathrm{p}}\Delta\rho_{\mathrm{p}}\right)^2$ and that of the ion-cloud signal is $s_{\mathrm{ic}} = 2V_{\mathrm{p}}\Delta\rho_{\mathrm{p}}\left(V_{\mathrm{ic}}^{\mathrm{NH_4}}\Delta\rho^{\mathrm{NH_4}} - V_{\mathrm{ic}}^{\mathrm{Na}}\Delta\rho^{\mathrm{Na}}\right)$. We obtain the ratio $s_{\mathrm{ic}}/s = 0.05 \pm 0.02$, where the uncertainty in the number of counterions per microgel gives the largest contribution to the error. The measured ratio, obtained from extrapolating the fits of the data to zero q, is $\Delta I_{\mathrm{corr}}(0)/I_{\mathrm{corr}}(0) = 0.06 \pm 0.01$, as mentioned in the main text. The agreement with the calculated ratio is very good and supports our interpretation that the signal due to the ions is a direct measurement of the counterion cloud.

At the lowest measured q, $I^{\mathrm{NH}_4^+}(q)$ is expected to have a slightly higher intensity than $I^{\mathrm{Na}^+}(q)$, as NH_4^+ has a larger contrast than Na^+ in the D_2O solvent. This is the case in all our measurements with the exception of sample s3 at $T \approx 45\,^\circ\mathrm{C}$ in the deswollen state for $q < 0.038$ nm^{-1}. In this q-range, the scattering signal is due to objects as large or larger than the whole microgel. Therefore, a small number of aggregates can explain the issue. At $T \geq T_{\mathrm{LCST}}$, the steric stabilization of microgels is weakened and some aggregation is possible [19]. As the s3 samples at $\zeta \approx 0.2$ are prepared by diluting the $\zeta = 0.55$ suspensions, it is reasonable that we observe a higher intensity of I^{Na^+} at low q's for both concentrations. In both cases, $\Delta I_{\mathrm{corr}}(q)$ is negative for $q < 0.038$ nm^{-1}, but the minimum due to the counterion cloud appears at a higher q and is still visible. We only use the data points at $q \geq 0.038$ nm^{-1} to extract the parameters of the counterion cloud. As shown in

Sample	$T(^\circ C)$ ± 0.1	ζ ± 0.04	$R_{\rm ic}$ (nm)	$\sigma_{\rm ic}$ (nm)	$R_{\rm SANS}$ (nm)
s1	20	0.10	83 ± 3	28 ± 4	83.9 ± 1.1
s1	20	0.47	72.4 ± 0.5	38 ± 4	78.8 ± 1.4
s2	20	0.11	123 ± 5	43 ± 17	124.5 ± 1.4
s2	20	0.48	117 ± 4	43 ± 21	118.7 ± 1.2
s2	45	0.11	51 ± 1	3 ± 3	49.5 ± 1.6
s2	45	0.48	50 ± 3	3 ± 2	49.4 ± 1.2
s3	20	0.17	111 ± 2	37 ± 2	134.3 ± 1.6
s3	20	0.55	110 ± 3	38 ± 5	128.8 ± 1.8
s3	45	0.17	52.5 ± 0.4	2 ± 1	54.6 ± 0.5
s3	45	0.55	51.8 ± 0.3	2.2 ± 0.3	55.2 ± 1.3
s3 with $\Delta I(q)/S'(q)$	20	0.17	112 ± 1	37 ± 3	134.3 ± 1.6
s3 New NH_4^+	20	0.17	112 ± 2	39 ± 2	135.0 ± 1.3

Supplementary Table 3: Results for the counterion cloud radius ($R_{\rm ic}$) and width ($\sigma_{\rm ic}$) obtained at $T \approx 20\,^\circ$C and $45\,^\circ$C using the model given in Eqs. 17, 18, and 19.

Supplementary Fig. 2D and I, the minimum is not as obvious as for $T \approx 20\,^\circ$C. However, as shown in the insets, the depth is of the same order as for the samples at $T = 20\,^\circ$C, despite being less visible over the whole q range due to the negative signal at the lowest q's; the cloud model captures the data nicely as shown in the insets of Supplementary Fig. 2D and I.

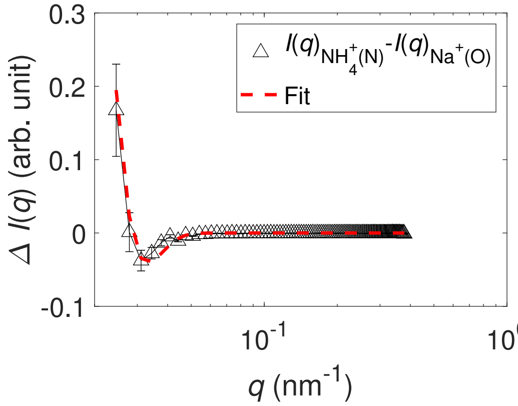

Supplementary Figure 4: Cloud signal and fit for s3 at $\zeta \approx 0.2$ with newly prepared NH_4^+ sample and old Na^+ sample. $\Delta I_{\rm corr}(q) = I_{\rm new}^{NH_4^+}(q) - I_{\rm old}^{Na^+}(q)$ with correction factor $n = 1.035$. The error bars represent the uncertainty due to the counting statistics of the SANS instrument.

For sample s2, the errors in $\sigma_{\rm ic}$ at $T = 20\,^\circ$C given in Supplementary Tab. 3 are larger than for samples s1 and s3. This is caused by the low resolution of the raw data and the limited q range. The magnitude of the error bars is comparable to the depth of the minimum and we are at the detection limit of the instrument SANS-II at SINQ that was used for these measurements; samples s1 and s3 were measured on SANS-I at SINQ. However, the minimum position is better defined and, as a result, reliable results are obtained for the cloud radius, $R_{\rm ic}$. At $T = 45\,^\circ$C, we find the cloud to

be centered close to the particle periphery for both $\zeta \approx 0.1$ and $\zeta \approx 0.5$. Due to the large errors in $\Delta I(q)$, Supplementary Fig. 2E and J, we fit the data with the limited q range $q \geq 0.035$ nm^{-1} around the minimum for the sample at $\zeta \approx 0.1$ and $q \geq 0.045$ nm^{-1} for the sample at $\zeta \approx 0.5$; the two lowest-q data points were excluded due to the large errors and because of large uncertainty in q. As the minimum is located at $q \approx 0.07$ nm^{-1}, the quality of the two lowest-q points does not affect the cloud signal. The results obtained with sample s2 are similar to those from sample s3, which is reassuring since the particle sizes are comparable and we expect analogous results.

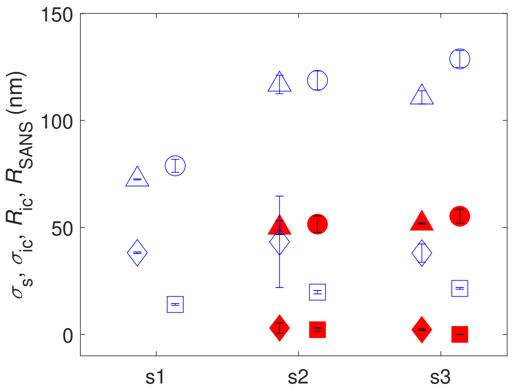

Supplementary Figure 5: The radii R_{SANS} ($\circ$) and R_{ic} ($\triangle$) as well as the half widths of the fuzzy corona, σ_{s}, ($\square$) and of the counterion cloud, σ_{ic}, ($\diamond$) for samples s1, s2, and s3 at $\zeta \approx 0.48$ measured at $T \approx 20\,^{\circ}$C (open symbols) and $T \approx 45\,^{\circ}$C (full symbols). All values shown are also listed in Supplementary Tabs. 2 and 3. The error bars result from the least-squares fit to the SANS data.

For sample s1 at $\zeta \approx 0.1$ and 0.5, the cloud model fits the $\Delta I(q)$ data shown in Supplementary Fig. 2C and H. The counterion cloud is found to be located close to R_{SANS} or slightly inside the fuzzy shell of the microgel. As for samples s3 and s2, σ_{ic} is about equal or slightly smaller than $2\sigma_{\mathrm{s}}$, that is, the width of the cloud is comparable to the thickness of the fuzzy corona. For sample s1 at $\zeta \approx 0.5$ and $q < 0.03$ nm^{-1}, Supplementary Fig. 2H, the fit lies above the measured data. The peak in the measured data at $q \approx 0.03$ nm^{-1} is probably due to a small difference in the structure factors of $I^{\mathrm{NH}_4^+}(q)$ and $I^{\mathrm{Na}^+}(q)$. $S'(q=0)$ is directly related to the compressibility and, therefore, the microgel density n_{d} of the suspension. As we find a small difference in n_{d} that we correct for in $I^{\mathrm{Na}^+}_{\mathrm{corr}}(q)$, this can explain a discrepancy at the lowest q's. As the fit works well around the minimum at $q \approx 0.05$ nm^{-1}, we obtain reliable parameters for the ion cloud. Further, we notice that R_{SANS} is smaller at $\zeta \approx 0.5$ compared to $\zeta \approx 0.1$, which implies that the true volume fraction at $\zeta \approx 0.5$ is $\phi \approx 0.4$, clearly below random close packing. This minor deswelling at $\zeta \approx 0.5$ is expected, as the counterion clouds are known to percolate at $\zeta \gtrsim 0.45$.

In addition, we have checked that the counterion cloud signal that we extract from the direct subtraction of $I^{\mathrm{NH}_4^+}(q)$ and $I^{\mathrm{Na}^+}(q)$ is a direct consequence of the ions in the sample and is repeatable. We have prepared a new s3 sample with NH_4^+ counterions from dry powder at $\zeta \approx 0.2$ and have measured it with SANS at $T = 20\,^{\circ}$C to compare it with the previously prepared NH_4^+ sample shown in Supplementary Fig. 1A. We then follow the same protocol to analyze the data and find that the

subtraction of the new and the old samples with NH_4^+ counterions does not have a minimum, see Fig. 2F in the main text. This is expected, as there is no contrast to detect the counterion cloud with NH_4^+ in both samples. As shown in Supplementary Fig. 4, we also find that the subtraction of the new NH_4^+ sample and the old Na^+ sample again shows the expected signal due to the counterion cloud, and we obtain the same radius and width within the accuracy of the analysis, see Supplementary Tab. 3.

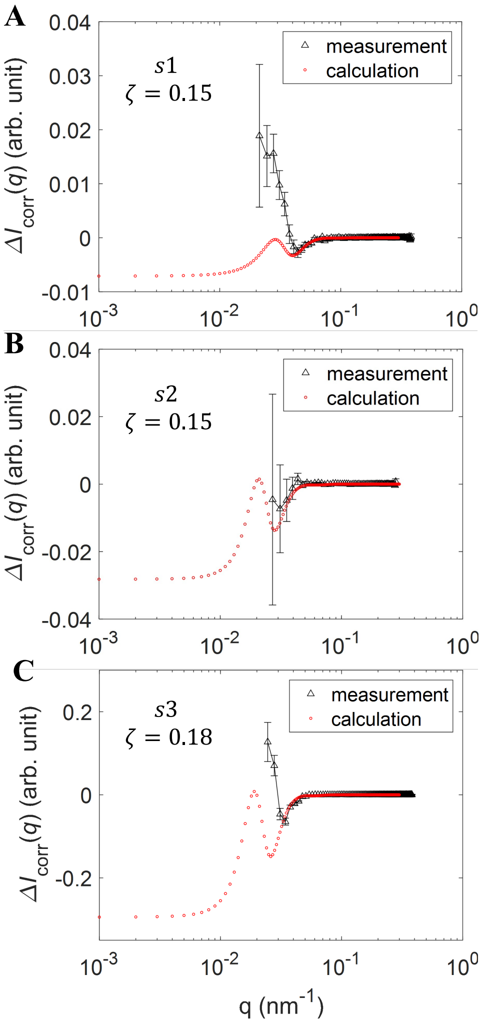

Supplementary Figure 6: Comparison of (△) the measured $\Delta I(q)$ signal and (∘) the calculated signal due to a small change of σ_s. The change of σ_s is adjusted to capture the higher-q part of the measured signal: (A) sample s1, $R_\mathrm{c} = 56$ nm and $\sigma_\mathrm{s} = 15$ nm for the larger particle and $\sigma_\mathrm{s} = 14.9$ nm for the smaller particle (B) sample s2, $R_\mathrm{c} = 82$ nm and $\sigma_\mathrm{s} = 21$ nm for the larger particle and $\sigma_\mathrm{s} = 20.9$ nm for the smaller particle and (C) sample s3, $R_\mathrm{c} = 87$ nm and $\sigma_\mathrm{s} = 24$ nm for the larger particle and $\sigma_\mathrm{s} = 23.2$ nm for the smaller particle. The error bars represent the uncertainty due to the counting statistics of the SANS instrument.

5 Effect of a possible structural change as modeled via changes in σ_s

To see the effect of changing the width of the fuzzy shell given by σ_s, as perhaps induced by the ions, we fix the core radius, R_c, and calculate $\Delta I(q) = I_\mathrm{large}(q) - I_\mathrm{small}(q)$, see Supplementary Fig. 6, where we subtract the $I(q)$ of the particle with smaller σ_s from the $I(q)$ with larger σ_s. As shown in Eqs. 7 and 17, a larger σ_s causes a faster decay of the microgel form factor with increasing q. Therefore, a negative minimum in the range $0.02\,\mathrm{nm}^{-1} < q < 0.05\,\mathrm{nm}^{-1}$ in $\Delta I(q)$ is obtained with the subtraction given above. The positive maximum close to $q = 0.01$ nm^{-1} is due to the structure factor that also depends on σ_s through the microgel radius $R_\mathrm{SANS} = R_\mathrm{c} + 2\sigma_\mathrm{s}$. We find that a change in σ_s in the range from 0.1 nm to 0.7 nm can give a minimum with the observed depth. While we can adjust the small difference in σ_s to reproduce the high-q side of the minimum in the experimental data, the low-q side of the observed minimum is not well reproduced, as the change in σ_s does not give the upturn observed in samples s1 and s3. As this upturn is quite pronounced, our ion-cloud model clearly provides a better fit to the data (see Fig. 2A in the main text and Supplementary Fig. 2A-C,F-H). Therefore, we find that the counterion-cloud model can reproduce all the observed data, while this is not possible with a change in the fuzzy shell thickness. This supports our interpretation of the observed $\Delta I(q)$ data with the counterion-cloud model.

The structural insensitivity to a change of counterions is further supported by studies on the influence that ions in the Hofmeister series have on the swelling behavior of pNIPAM [20]. A significant effect on the swelling behavior of pNIPAM, as well as a significant difference between ion types, was only found at high ion concentrations, $\gtrsim$ 300 mM. The counterion concentration in the samples we study is well below this concentration. We estimate the maximum counterion concentration in our samples by assuming that $N = 5 \cdot 10^4$ counterions are distributed in the fuzzy shell of the microgel and obtain $N/[N_\mathrm{A}\, 4\pi(R_\mathrm{c} + \sigma_\mathrm{s})^2(2\sigma_\mathrm{s})] \approx 16$ mM. Here, we use the middle radius $R_\mathrm{c} + \sigma_\mathrm{s}$ and the thickness $2\sigma_\mathrm{s}$ of the fuzzy corona; N_A is Avogadro's number. Consistent with this, additional work on the Hofmeister series also finds that a salt concentration of ~ 10 mM has negligible effects on the lower critical solution temperature (LCST) of pNIPAM [21]. The low ion concentration in our ion clouds and the fact that our SANS measurements do not show a structural difference between samples with NH_4^+ and Na^+ ions provide further support for the presented analysis of the counterion clouds of the microgels.

References

[1] J. Clark, *Reactions of alkenes with sulfuric acid*, https://chem.libretexts.org, 2020.

[2] U. Gasser, A. Scotti, and A. Fernandez-Nieves, "Spontaneous deswelling of microgels controlled by counterion clouds", Phys. Rev. E **99**, 042602 (2019).

[3] L. Zhang, E. S. Daniels, V. L. Dimonie, and A. Klein, "Synthesis and characterization of pnipam/ps core/shell particles", Journal of applied polymer science **118**, 2502–2511 (2010).

[4] G. Romeo, L. Imperiali, J.-W. Kim, A. Fernandez-Nieves, and D. A. Weitz, "Origin of deswelling and dynamics of dense ionic microgel suspension", J. Chem. Phys. **136**, 124905 (2012).

[5] G. Batchelor, "The effect of brownian motion on the bulk stress in a suspension of spherical particles", Journal of fluid mechanics **83**, 97–117 (1977).

[6] T. Kyrey, J. Witte, J. Lutzki, M. Zamponi, S. Wellert, and O. Holderer, "Mobility of bound water in pnipam microgels", Phys. Chem. Chem. Phys. **23**, 14252–14259 (2021).

[7] M. Stieger, J. Pedersen, P. Lindner, and W. Richtering, "Are thermoresponsive microgels model systems for concentrated colloidal suspensions? a rheology and small-angle neutron scattering study", Langmuir **20**, 7283–7292 (2004).

[8] T. Eckert and W. Richtering, "Thermodynamic and hydrodynamic interaction in concentrated microgel suspensions: hard or soft sphere behavior?", J. Chem. Phys. **129**, 124902 (2008).

[9] A. Scotti, U. Gasser, E. S. Herman, J. Han, A. Menzel, L. A. Lyon, and A. Fernandez-Nieves, "Phase behavior of binary and polydisperse suspensions of compressible microgels controlled by selective particle deswelling", Phys. Rev. E **96**, 032609 (2017).

[10] M. Kotlarchyk and C. Sow-Hsin, "Analysis of small angle neutron scattering spectra from polydisperse interacting colloids", J. Chem. Phys. **79**, 2461–2469 (1983).

[11] N. Ashcroft and D. C. Langreth, "Structure of binary liquid mixtures. i", Physical Review **156**, 685 (1967).

[12] M. Stieger, W. Richtering, J. Pedersen, and P. Lindner, "Small-angle neutron scattering study of structural changes in temperature sensitive microgel colloids", J. Chem. Phys. **120**, 6197–6206 (2004).

[13] X. Wu, R. Pelton, A. Hamielec, D. Woods, and W. McPhee, "The kinetics of poly(n-isopropylacrylamide) microgel latex formation", Colloid and Polymer Science **272**, 467–477 (1994).

[14] A. Fernandez-Barbero, A. Fernandez-Nieves, I. Grillo, and E. Lopez-Cabarcos, "Structural modifications in the swelling of inhomogeneous microgels by light and neutron scattering", Physical Review E **66**, 051803 (2002).

[15] K. Kratz, T. Hellweg, and W. Eimer, "Structural changes in pnipam microgel particles as seen by sans, dls, and em techniques", Polymer **42**, 6631–6639 (2001).

[16] J. S. Pedersen, D. Posselt, and K. Mortensen, "Analytical treatment of the resolution function for small-angle scattering", Journal of Applied Crystallography **23**, 321–333 (1990).

[17] A. Scotti, U. Gasser, E. S. Herman, M. Pelaez-Fernandez, L. A. Lyon, and A. Fernandez-Nieves, "The role of ions in the self-healing behavior of soft particle suspensions", Proc. Natl. Acad. Sci. USA **113**, 5576–5581 (2016).

[18] U. Gasser, A. Scotti, and A. Fernandez-Nieves, "Spontaneous deswelling of microgels controlled by counterion clouds", Physical Review E **99**, 042602 (2019).

[19] M. Stieger, J. S. Pedersen, P. Lindner, and W. Richtering, "Are thermoresponsive microgels model systems for concentrated colloidal suspensions? a rheology and small-angle neutron scattering study", Langmuir **20**, 7283–7292 (2004).

[20] T. Lopez-Leon and A. Fernandez-Nieves, “Macroscopically probing the entropic influence of ions: deswelling neutral microgels with salt”, Phys. Rev. E **75**, 011801 (2007).

[21] B. Kang, H. Tang, Z. Zhao, and S. Song, “Hofmeister series: insights of ion specificity from amphiphilic assembly and interface property”, ACS Omega **5**, 6229–6239 (2020).
